# A factor model to analyze heterogeneity in gene expression

**DOI:** 10.1186/1471-2105-11-368

**Published:** 2010-07-02

**Authors:** Yuna Blum, Guillaume Le Mignon, Sandrine Lagarrigue, David Causeur

**Affiliations:** 1 Agrocampus Ouest, UMR598, Animal Genetics, 35000 Rennes, France; 2 INRA, UMR598, Animal Genetics, 35000 Rennes, France; 3 Agrocampus Ouest, Applied Mathematics Department, 35000 Rennes, France

## Abstract

**Background:**

Microarray technology allows the simultaneous analysis of thousands of genes within a single experiment. Significance analyses of transcriptomic data ignore the gene dependence structure. This leads to correlation among test statistics which affects a strong control of the false discovery proportion. A recent method called FAMT allows capturing the gene dependence into factors in order to improve high-dimensional multiple testing procedures. In the subsequent analyses aiming at a functional characterization of the differentially expressed genes, our study shows how these factors can be used both to identify the components of expression heterogeneity and to give more insight into the underlying biological processes.

**Results:**

The use of factors to characterize simple patterns of heterogeneity is first demonstrated on illustrative gene expression data sets. An expression data set primarily generated to map QTL for fatness in chickens is then analyzed. Contrarily to the analysis based on the raw data, a relevant functional information about a QTL region is revealed by factor-adjustment of the gene expressions. Additionally, the interpretation of the independent factors regarding known information about both experimental design and genes shows that some factors may have different and complex origins.

**Conclusions:**

As biological information and technological biases are identified in what was before simply considered as statistical noise, analyzing heterogeneity in gene expression yields a new point of view on transcriptomic data.

## Background

Microarray technology allows the analysis of expression levels for thousands of genes simultaneously and is a powerful tool to characterize mRNA level variation due to measured variables of interest (various phenotypes, treatments...). Typical approaches to find significant relationships between gene expressions and experimental conditions ignore the correlations among expression profiles and functional categories [1]. This dependence structure leads to correlation among test statistics which affects a strong control of the actual proportion of false discoveries [2]. Indeed, a number of unmeasured or unmodeled factors independent of the variables of interest may influence the expression of any particular gene [3,4]. These factors may induce extra variability in the expression levels and decrease the power to detect links with the variables of interest.

Recently, several works have introduced models for the common information shared by all the genes. Especially Friguet *et al *[4] propose to model this sharing of information by a factor analysis structure in a method called Factor Analysis for Multiple Testing (FAMT). The estimated factors in the model capture components of the expression heterogeneity. As well, Storey *et al *[3] introduce Surrogate Variable Analysis (SVA) to identify and estimate these extra sources of variation. The factors in FAMT and the surrogate variables in SVA are similarly designed to model dependence among tests by a linear kernel but they are estimated differently. Contrarily to the SVA model, independence between the factors and the experimental conditions of interest is explicitly assumed in FAMT in order to separate clearly the effects of the experimental conditions on the gene expressions and the nuisance variability due to unmodeled technological effects and other known or unknown effects that could be uncontrolled in the experimental design.

The major sources of expression variation are then assumed to be the experimental conditions of interest, but also gene dependence and uncontrolled factors in the experimental design. Indeed, even after normalization, variation due to the experimental design still exists in expression data. The factors extracted in the residual part of the regression models explaining the gene expressions by the experimental conditions of interest are therefore analyzed to give more insight both on expression heterogeneity among sampling units and the contribution of some biological processes to gene dependence. First, factors are extracted from illustrative expression data sets with simple patterns of expression heterogeneity in order to show how they can straightforward be related to sources of heterogeneity. Henceforth, the same factor model approach is used to analyze an expression data set initially generated to map quantitative trait loci (QTL) for abdominal fatness (AF) in chickens, especially on chromosome 5 (GGA5) [5]. This data set concerns hepatic transcriptome profiles for 11213 genes of 45 half sib male chickens generated from a same sire. This sire was generated by successive inter-crossing of two experimental chicken lines divergently selected on AF and was known to be heterozygous for an AF QTL on the GGA5 chromosome around 175 cM (For more details, see [5]). The 45 half sib chickens show therefore variation on AF. According to the polygenic effect model of quantitative traits, this variation is probably due to multiple mutations and biological processes.

Two lists of genes significantly correlated to the AF trait are first generated using the raw and the factor-adjusted expression dataset. Then, the relevance of the two gene lists to characterize functionally fatness variation in the family are compared, regarding the frequencies of biological processes related to the AF trait in their functional annotations. Factor-adjusted expression data is finally used to identify a gene whose expression is controlled by the AF QTL region.

Furthermore, the extracted factors are interpreted using external information on the experimental design such as the hatch, dam and body weight and also gene information such as functional categories, oligonucleotide size and location on the microarray. It is deduced that some factors may have different and complex origins, which confirms the importance of taking into account these extra sources of variability to be more relevant in the transcriptomic analyses.

## Results

### Illustrative Examples

Similarly to Storey *et al *(2007) [3], three simple situations of heterogeneity are considered. For each one, independent expressions for 1000 genes on 20 arrays are simulated according to a standard normal distribution. The sample is split into two equal groups and a constant is added on the first 100 gene expressions to mimic a differential expression between these two groups.

#### Case 1: One independent variable affecting all genes

**Figure 1:**
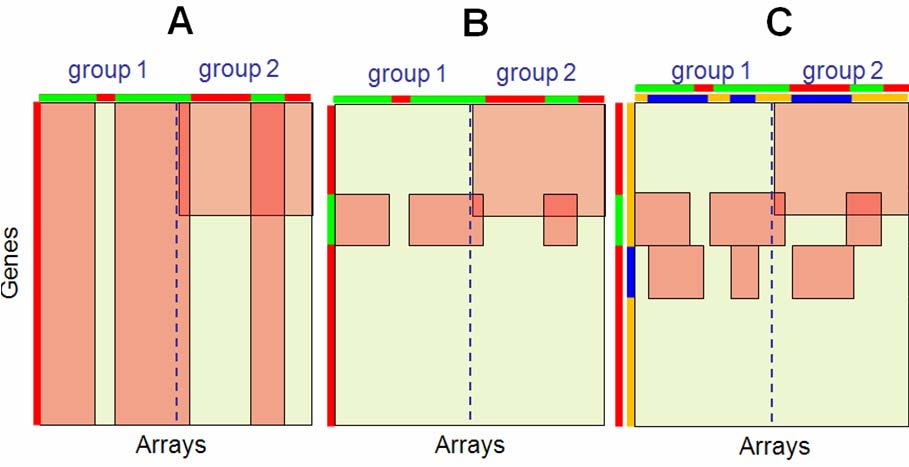
**Structure of the illustrative data sets**. Representation of three illustrative studies consisting of 1000 genes on 20 arrays divided between two groups. (A) Case 1: one independent grouping variable with red and green levels affecting all genes. (B) Case 2: one independent grouping variable with red and green levels affecting a gene set. (C) Case 3: two independent grouping variables with red and green levels and with blue and orange levels affecting each a different gene set.

**Figure 2:**
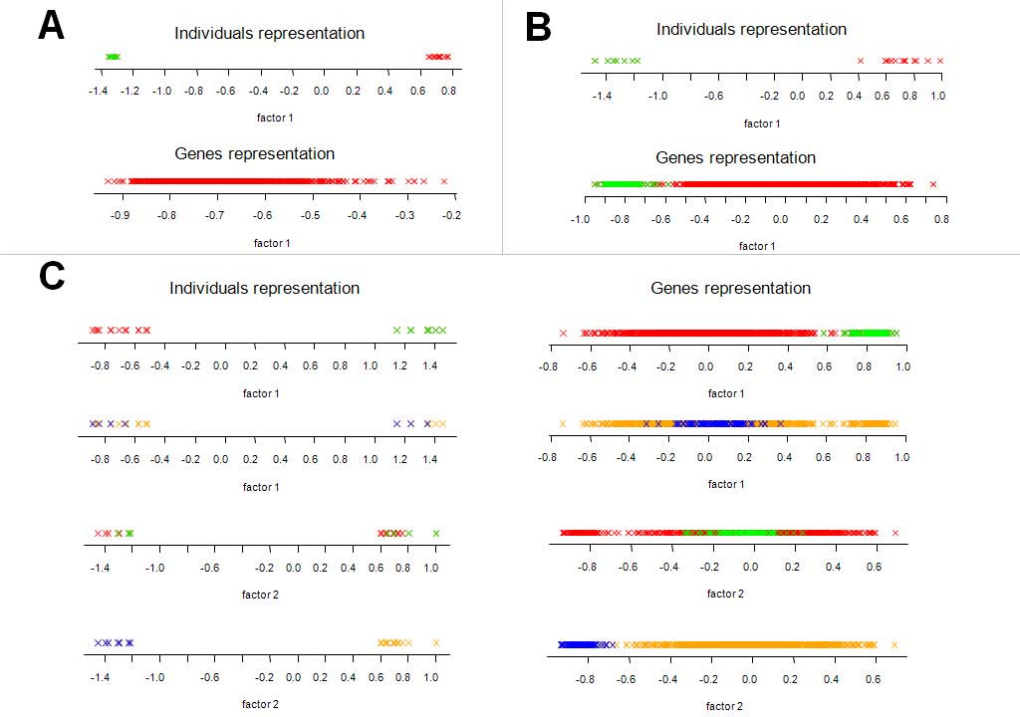
**Illustrative data sets: Individuals and genes representations**. Individuals and genes representation using respectively the Z matrix corresponding to the factors and the B matrix of the loadings found by FAMT. Individuals and genes are colored according to the independent variable they are affected by. (A) representations corresponding to case 1, (B) case 2 and (C) case 3.

All genes are affected by an independent grouping variable marked by colors red and green on Figure 1A. A single factor is extracted by FAMT. Figure 2A helps interpreting this factor and shows that it clearly discriminates the two colored groups of individuals (P-value ≤ 2.2 × 10^-16^). This shows a high association between the factor and the independent grouping variable. The genes representation does not show any particular structure. In this simple case the factor estimated by FAMT can therefore be easily interpreted through the individuals representation.

#### Case 2: One independent variable affecting a set of genes

Only genes 70-170 are affected by an independent grouping variable marked by colors red and green on Figure 1B. A single factor is also found using FAMT. As shown on Figure 2B, the factor discriminates the two groups of individuals (P-value ≤ 2.2 × 10^-16^) and the two groups of genes (P-value ≤ 2.2 × 10^-16^). In this case, the estimated factor can be interpreted through the individuals and genes representations.

#### Case 3: Two independent variables affecting two different sets of genes

Gene sets 70-170 and 171-271 are each affected by an independent grouping variable marked respectively by colors red and green and by colors orange and blue as illustrated by Figure 1C. Two factors are identified by FAMT which are now interpreted regarding the two external sources of heterogeneity (Figure 2C). The red-green variable seems to be highly associated with the first axis (P-value ≤ 2.2 × 10^-16 ^in both representation). On the contrary, the orange-blue variable is not associated with this axis considering a significance level of 0.05 (P-value = 0.7933 for the individuals representation, p-value = 0.1109 for the genes representation). The same strategy is implemented for the second factor. The red-green variable appears to be not associated with this factor (P-value = 0.7949 for the individuals representation, p-value = 0.1926 for the genes representation) whereas the orange-blue variable is highly associated (P-value ≤ 2.2 × 10^-16 ^in both representations). In this case, each of the two estimated factors can be explained by one of the two independent grouping variables.

### Analysis of the AF expression data set

#### Classical approach

Examination of the Pearson coefficient correlation between hepatic transcript levels and AF trait shows that 287 genes are significantly correlated considering a significance threshold of 0.05 without any correction for multiple tests. This low amount of differentially expressed genes might be explained by a poor genetic variability between individuals which are half sib offsprings and could also be due to dependence between genes that can lead to under representation of the smallest p-values [6].

#### Heterogeneity analysis

**Figure 3:**
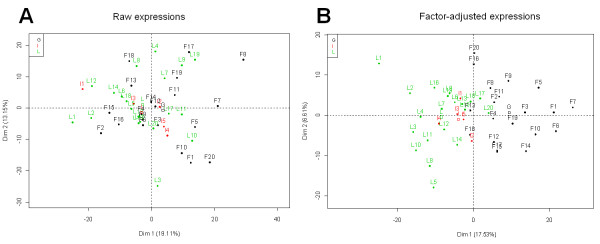
**Principal component analysis: individuals representation**. Birds are colored according to their weight split into 3 classes: lean (green), intermediate (red) and fat (black). (A) PCA generated with the raw expression of the 688 differentially expressed genes. (B) PCA generated with the factor-adjusted expression of the 688 differentially expressed genes.

Minimizing the variance inflation criterion proposed by Friguet *et al*. [4], six factors containing a common information shared by all genes and independent from the AF trait are extracted. Subtracting the linear dependence kernel defined by these factors from the raw expression data yields the factor-adjusted expression data. The significance analysis based on these expressions results in a list of 688 gene expressions significantly correlated to the AF trait. 93% of the 287 genes found with the classical approach are included in this list. This larger number of differentially expressed genes suggests that correlation between many gene expressions and the variable of interest is under estimated due to gene dependence. Considering the Gene Ontology (GO) terms and KEGG pathways, one enriched term related to the lipid metabolism is found in the gene list resulting from factor-adjustment (688 genes) whereas none is observed in the gene list obtained using the raw expressions (287 genes). This term concerns "Steroid biosynthesis process" with 3 genes associated (Table 1). More precisely, these genes are involved in the cholesterol metabolism or in conversion of cholesterol in steroids. Several works show relationships between cholesterol metabolism and obesity [7-9]. This result shows that the genes found after factor-adjustment are more related to the fatness trait. Furthermore, the impact of factor-adjustment is shown in Figure 3, where a principal component analysis (PCA) generated with the 688 factor-adjusted transcript levels of correlated genes (Figure 3B) separates much more fat and lean chickens than the same PCA generated with the raw expressions of the same 688 genes (Figure 3A). This observation displays that factor-adjustment has cleaned up the data from dependence, which highlights masked relationships with the AF trait.

**Table 1:** Enrichment tests for the list of 287 genes and 688 genes

LIST OF 287 GENES					
**GOID**	**GO Term**	**Size**	**Count**	**Pvalue**	**HGNC ID**

GO.0006470	*protein amino acid dephosphorylation*	56	5	0.015	ACP1, PTPN14, PTPRE, PTP4A3, PTPN6
GO.0006725	*cellular aromatic compound metabolic process*	38	4	0.017	PPME1, GART, MOCS1, ALDH6A1
GO.0007259	*JAK STAT cascade*	9	2	0.022	SOCS1, STAMBP
GO.0043543	protein amino acid acylation	9	2	0.022	NULL, ZDHHC17
GO.0044259	multicellular macromolecule metabolic process	10	2	0.027	ACE2, SERPINH1
GO.0008033	tRNA processing	26	3	0.0296	TSEN15, FARS2, NSUN2
GO.0033002	muscle cell proliferation	11	2	0.032	NOX1, BMP10
GO.0050730	regulation of peptidyl tyrosine phosphorylation	12	2	0.038	SOCS1, EGFR

Kegg ID	Kegg pathway	Size	Count	Pvalue	HGNC ID

map04320	*Dorso ventral axis formation*	9	3	2.38E-03	EGFR, SPIRE1, ETS1

LIST OF 688 GENES					

GOID	GO Term	Size	Count	Pvalue	HGNC ID

GO.0006470	*protein amino acid dephosphorylation*	56	10	1.80E-03	ACP1, PPM1E, PTPN14, PTPRE, PTP4A3, PPM1G, PTPRU, PPP3CB, PPM1L, PTPRF
GO.0046483	heterocycle metabolic process	33	7	3.21E-03	AMBP, GART, P4HA2, HMOX2, AFMID, MTHFS, ALDH6A1
GO.0051186	cofactor metabolic process	64	10	4.97E-03	AMBP, TXNRD3, NOX1, HMOX2, AFMID, GGT7, MTHFS, MOCS1, HMGCS1, ACO2
GO.0016202	regulation of striated muscle development	15	4	0.011	MBNL3, LEF1, NRG1, BMP4
GO.0007259	*JAK STAT cascade*	9	3	0.014	SOCS1, HCLS1, STAMBP
GO.0040011	locomotion	111	13	0.017	PRKG1, EDNRB, ACE2, NOX1, EGFR, NRG1, BMP10, ARAP3, JPH3, VHL, VAX1, DAB1, LAMA2
GO.0001932	regulation of protein amino acid phosphorylation	26	5	0.019	PDGFA, SOCS1, HCLS1, EGFR, BMP4
GO.0048585	negative regulation of response to stimulus	10	3	0.020	AMBP, PPP3CB, FABP7
GO.0006534	cysteine metabolic process	4	2	0.021	CBS, CDO1
GO.0002274	myeloid leukocyte activation	11	3	0.026	IRF4, LCP2, NDRG1
GO.0006725	*cellular aromatic compound metabolic process*	38	6	0.026	PPME1, GART, AFMID, MTHFS, MOCS1, ALDH6A1
GO.0007185	transmembrane receptor tyrosine phosphatase signaling	5	2	0.033	PTPRE, PTPRF
GO.0007271	synaptic transmission cholinergic	5	2	0.033	CHRNA4, LAMA2
GO.0000097	sulfur amino acid biosynthetic process	5	2	0.033	CBS, CDO1
GO.0006700	**C21 steroid hormone biosynthetic process**	5	2	0.033	STAR, CYP17A1
GO.0006787	porphyrin catabolic process	5	2	0.033	AMBP, HMOX2
GO.0001764	neuron migration	12	3	0.033	PRKG1, VAX1, DAB1
GO.0030509	BMP signaling pathway	21	4	0.036	SOSTDC1, BMP10, MSX2, BMP4
GO.0045321	leukocyte activation	64	8	0.040	SWAP70, CHRNA4, FKBP1B, IRF4, LCP2, PPP3CB, NDRG1, SFRS17A
GO.0006790	sulfur metabolic process	32	5	0.043	CBS, CDO1, TXNRD3, GGT7, CHST1
GO.0018193	peptidyl amino acid modification	43	6	0.045	PDGFA, SOCS1, P4HA2, HCLS1, EGFR, MAP2
GO.0008211	glucocorticoid metabolic process	6	2	0.048	STAR, CYP17A1
GO.0006769	nicotinamide metabolic process	6	2	0.048	NOX1, AFMID
GO.0030111	regulation of Wnt receptor signaling pathway	14	3	0.050	SENP2, LEF1, SENP2

Kegg ID	Kegg pathway	Size	Count	Pvalue	HGNC ID

map00630	Glyoxylate and dicarboxylate metabolism	9	4	1.87E-03	GLYCTK, HYI, AFMID, ACO2
map00140	**C21 Steroid hormone metabolism**	6	3	5.11E-03	**DHCR7**, HSD11B1, CYP17A1
map04320	*Dorso ventral axis formation*	9	3	0.018	EGFR, SPIRE1, ETS1
map04012f	ErbB signaling pathway	35	6	0.026	PIK3R5, PLCG1, PAK3, EGFR, NRG1, PTK2

Enrichment tests were performed using an R program (see Methods section) with GO BP terms and Kegg pathways. The tests were done on the list of 287 genes found using the classical approach and the list of 688 genes found by FAMT. For each enriched term, the identifier (ID), the biological term, the size of the whole list of genes related to the term (size), the number of genes in the sub-list related to the term (count), the pvalue of the test and the HGNC Hugo abbreviations of the related genes are given. Italic terms are those which are present in both lists.

**Figure 4:**
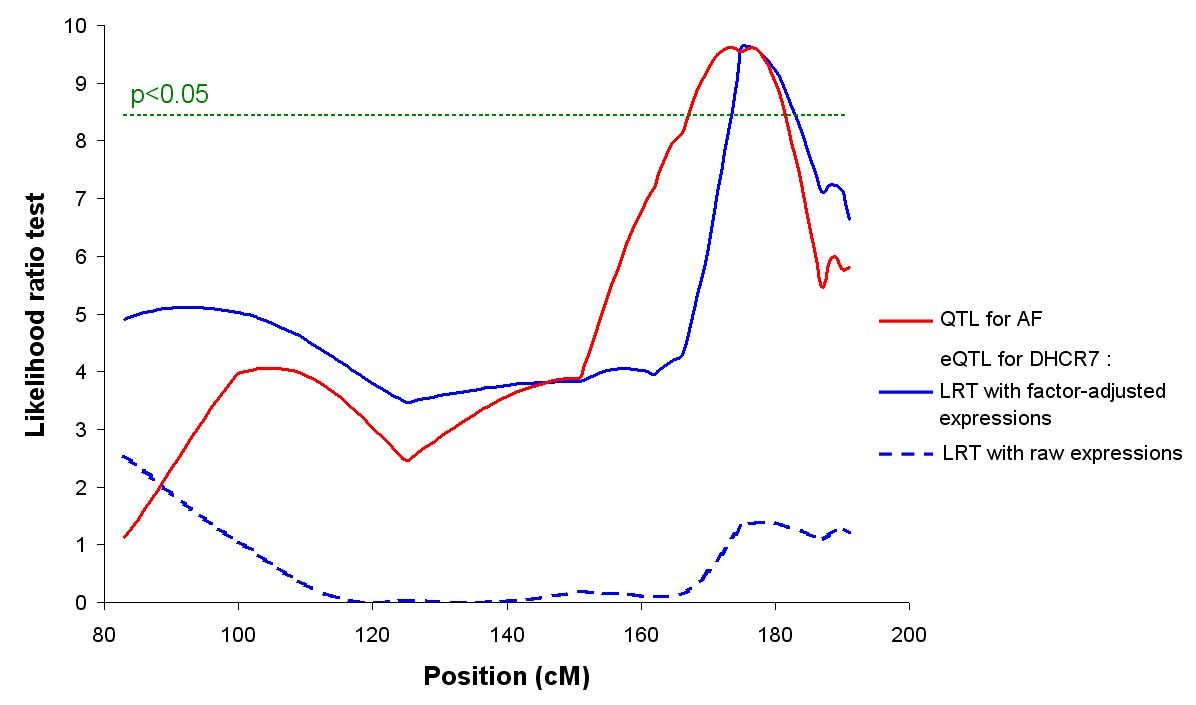
**eQTL mapping for DHCR7 on chromosome 5**. The LRT curves for the gene DHCR7 are represented in blue (plain line for the factor-adjusted analysis and dotted line for the raw analysis). The LRT curve for the AF trait is represented in red. The plain curves reveal the existence of a QTL/eQTL for the 2 traits in the same region, around 175 cM on the GGA5 chromosome. This colocalization is not revealed by the raw analysis. The Significance level of 5% is represented by the horizontal green line. The genetic distances (cM) and likelihood ratio (LR) are shown on the X-axis and Y-axis, respectively.

We focus on one of the 3 genes involved in the "Steroid biosynthesis process", DHCR7, which is only observed in the list of 688 genes and known for encoding the last enzyme involved in the cholesterol synthesis. As shown in Figure 4, the analysis of the factor-adjusted expressions for this gene highlights an eQTL (P-value < 0.05) colocalizing with the AF QTL previously observed [5]. The same LRT curve based on the raw expressions does not point out any eQTL. This result shows that the expression of this gene is controlled by a mutation in the same GGA5 AF QTL region. Because of the function of this gene related to lipid metabolism, this result suggests that this mutation could be the same as the QTL mutation for fatness phenotype. Further investigations are necessary to refine these QTL and eQTL locations.

### Factor interpretation

In the present study, some external information about the experimental design and the genes is available. As we did in the simulated examples, we interpret the factors extracted from the AF expression dataset using this known information.

#### Using information on experimental design

**Figure 5:**
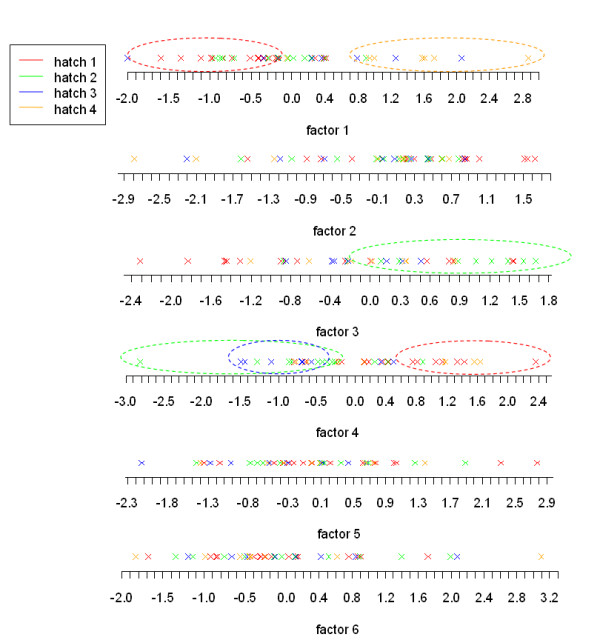
**AF data set: individuals representation for each factor**. Individuals are represented on the factor using the B matrix of loadings found by FAMT. The individuals are colored according to the variable "hatch" which has 4 levels.

The hatch, the dam and the body weight were previously measured for each bird and should be independent of the AF variation. For the body weight, the founder chicken lines were selected on AF criteria maintaining a constant body weight. The variables "hatch" and "dam" are both categorical with respectively, four and eight levels and the body weight is a continuous variable. We first focus on the "hatch" and for each factor we represent the individuals colored according to their hatch (Figure 5). Factor 1 seems to discriminate hatches 1 and 4, factor 3 hatch 2 from the others and factor 4 hatches 2 and 3 from hatch 1. The effect of the hatch on each factor is tested and the results given in Table 2 confirm our previous observations: factor 1, 3 and 4 can be partly explained by a hatch effect (the significant test for each hatch level is given in Additional file 1). We then calculate the association for the "dam" and "body weight" with each of the six factors. Table 2 shows no effect of the dam and a high correlation between the weight and factor 2. Contrarily to the illustrative cases where each factor could be interpreted by a unique variable, the factors found here seem to have more complex origins. Indeed, three of the six factors can be interpreted by an hatch effect and another one by a body weight effect. The same analysis is now performed after adjustment of the raw expression data for hatch and body weight. Interestingly, only five factors independent of the AF trait are extracted and still a hatch effect exists but only on the first factor and a weight effect on the second factor (Table 2). This persistence of both effects suggests that there exists an interaction involving hatch and body weight with other unmeasured and/or unknown variables. Therefore, taking into account the hatch and body weight in the statistical model seems to be not sufficient to remove a consequent part of the heterogeneity in gene expression.

**Table 2:** Description of the factors extracted from the raw data and from the hatch and weight adjusted data

Factors extracted from the raw AF expression dataset							
	**Individual information**			**Gene information**			
	
	**hatch**	**dam**	**weight**	**oligo size**	**chip block**	**chip row**	**chip column**

Factor 1	**8.92E-05**	0.139	0.129	**2.20E-16**	**2.20E-16**	0.074	0.179
Factor 2	0.074	0.913	**4.70E-03**	**2.20E-16**	**2.20E-16**	0.041	0.857
Factor 3	**1.90E-02**	0.848	0.489	**2.55E-14**	**2.20E-16**	0.716	0.376
Factor 4	**6.00E-03**	0.127	0.959	**1.41E-07**	**2.20E-16**	0.707	0.167
Factor 5	0.435	0.217	0.884	0.529	**2.20E-16**	**4.97E-03**	**9.99E-05**
Factor 6	0.946	0.412	0.615	**1.79E-07**	**2.20E-16**	0.876	**5.11E-07**

**Factors extracted from the AF expression dataset adjusted for the hactch and body weight effects**							

	**Individual information**			**Gene information**			
	
	**hatch**	**dam**	**Weight**	**oligo size**	**chip block**	**chip row**	**chip column**

Factor 1	**1.13E-04**	0.219	0.156	**2.20E-16**	**2.20E-16**	0.078	0.209
Factor 2	0.052	0.841	**3.40E-03**	**2.20E-16**	**2.20E-16**	0.036	0.814
Factor 3	0.049	0.819	0.569	**2.16E-11**	**2.20E-16**	0.554	0.16
Factor 4	0.178	0.031	0.869	**6.80E-09**	**2.20E-16**	0.897	0.885
Factor 5	0.949	0.727	0.647	**2.79E-12**	**2.20E-16**	0.291	**2.36E-10**

The p-value is given for each association test (see Methods section). Considering a threshold of 1%, the significant p-values are in bold.

#### Using gene information

To interpret the estimated factors in terms of gene expressions, we use known information about genes as oligonucleotide size and location on the chip: block, row and column (genes representation on each factor is given in Additional file 2). For these variables, we test their association with each factors extracted from the raw data and the hatch and body weight adjusted data. As shown in Table 2, there is a strong oligonucleotide size effect and block effect captured by almost all the factors. We exhibit also a row and column effect associated with some factors. Moreover, the genes that most contribute to the first two factors are identified (score larger than 0.8). We obtain a set of 313 genes for factor 1 and a set of 175 genes for factor 2 and which were as expected not included for 95% of them in the list of 688 genes. We perform a term enrichment test for this two sets (Table 3). As we expect, there are essentially biological terms independent of the lipid metabolism. Factor 1 is mainly characterized by genes involved in cell division metabolism and interestingly also to pigmentation. Factor 2 is more characterized by genes involved in the nucleotide metabolism. The enriched terms found are thus not implicated in the metabolim changes induced by the AF variability. We previously highlighted a hatch and body weight effects on the factors. As in PCA, individuals and genes representations can be interpreted commonly. Hatch effect could therefore be related to the particular metabolisms characterizing factor 1.

**Table 3:** Biological terms characterizing factor 1 and 2

FACTOR1				
**GO ID**	**GO Term**	**Size**	**Count**	**Pvalue**

GO.0007051	spindle organization	6	2	8.20E-03
GO.0050931	pigment cell differentiation	7	2	0.011
GO.0000279	M phase of meiotic cell cycle	79	6	0.012
GO.0000079	regulation of cyclin dependent protein kinase activity	9	2	0.019
GO.0016570	histone modification	13	2	0.038
GO.0015698	inorganic anion transport	53	4	0.039
GO.0007156	homophilic cell adhesion	32	3	0.041

Kegg ID	Kegg pathway	Size	Count	Pvalue

map05216	Thyroid cancer	11	2	0.020
map05130	Pathogenic Escherichia coli infection	12	2	0.024
map04520	Adherens junction	31	3	0.024

FACTOR2				

GO ID	GO Term	Size	Count	Pvalue
GO.0006195	purine nucleotide catabolic process	5	3	2.23E-05
GO.0030168	platelet activation	7	3	7.65E-05
GO.0007051	spindle organization	6	2	2.55E-03
GO.0007596	blood coagulation	24	3	3.76E-03
GO.0030336	negative regulation of cell migration	12	2	0.011
GO.0032879	regulation of localization	103	5	0.012
GO.0001775	cell activation	74	4	0.016
GO.0001890	placenta development	18	2	0.023
GO.0017038	protein import	49	3	0.027
GO.0006403	RNA localization	22	2	0.034
GO.0006816	calcium ion transport	56	3	0.038

Kegg ID	Kegg pathway	Size	Count	Pvalue

map00230	Purine metabolism	64	4	0.013

Enrichment tests were performed on the genes contributing the more to the construction of factor 1 and 2 using the GO BP terms and Kegg pathways. For each enriched term, the identifier (ID), the bioligical term, the size of the whole list of genes related to the term (size), the number of genes in the sub-list related to the term (count), the pvalue of the test.

## Discussion and Conclusion

The model used in the present study assumes that the gene expressions are uncorrelated given a set of hidden variables called *factors*. In comparison to classical methods which do not take into account the dependence between genes, this approach provides a list of genes more correlated to the variable of interest. Moreover, factor-adjustment of the expression dataset turns out to give more insight to subsequent analyses such as QTL characterization. As a result, a gene is identified as correlated to the AF trait and related to the cholesterol metabolism having a trans-eQTL colocalizing with GGA5 AF QTL. Because several works show a link between cholesterol and obesity, this gene could be considered as a signature of the mutation underlying this AF QTL rather than a mutation close to it. This result provides functional hypothesis about genes whose expression could be impacted by the QTL of interest.

Factor analysis was introduced in the psychometric field in 1904 by Spearman [10] in order to extract the common factors in intelligence and personality. In this particular domain, the individuals are explained by their responses to different subsets of tests. The method usually furnished at least five factors which were interpreted as follows: neuroticism, extraversion, conscientiousness, agreeableness and openness to ideas. In our study, the factors were found using an EM algorithm presented by [4]. Our purpose was first to interpret the estimated factors and consequently to investigate which kind of information present in this factor structure could generate heterogeneity of the gene expressions. External information concerning the experimental design and functional annotations of the genes were used to analyse the factors. It is deduced that some factors seem to have a complex explanation with at least 2 variables associated to them. For factor 1, the individuals variability independent of the trait of interest is for instance shown to be related to the hatch. Enrichment tests also give a characterization of this factor by specific metabolisms.

To remove expression heterogeneity from the data for the subsequent statistical analyses, the basic idea consists in adjusting the raw expression data from the common factor structure. As we extract uncontrolled effects and technological biases from what was before simply considered as statistical noise, analyzing heterogeneity in gene expression yields a new point of view on transcriptomic data. We show in this study the importance of taking into account these extra sources of variation to be more relevant in the transcriptomic analyses.

## Methods

### AF expression data set

The data set concerns hepatic transciptome profiles for 11213 genes of 45 half sib male chickens variable for abdominal fatness (AF). The data set was generated to map quantitative trait loci (QTL) for abdominal fatness in chickens and used in a previous study [5]. The sire of this family, generated by successive inter-crossing of two experimental chicken lines divergently selected on abdominal fatness, was known to be heterozygous for an AF QTL on the GGA5 chromosome around 175 cM. Animals, marker genotyping and transcriptome data acquisition and normalization are described in Le Mignon *et al *(2009) [5].

### Illustrative examples

For each case, we simulated expression for 1000 genes on 20 arrays divided in two groups using the R programming language. Initially, the expression measurements for each gene were independently drawn from a standard normal distribution. The expression heterogeneity due to simple independent grouping variables was included in the simulated data set by adding a constant value for 7 random individuals for all genes (case 1) or a set of genes (case 2 and 3).

### Classical expression analysis

As the variable of interest in the biological study is continuous, we calculated the Pearson correlation coefficient for each gene expression and deduced the number of genes correlated to the trait by considering the P-values under the cutoff 0.05.

### Factor-analytic method

#### Steps and algorithm

The method takes into account the impact of dependence on the multiple testing procedures for high-throughput data. The common information shared by all the variables (i.e. gene expressions) is modeled by a factor analysis structure. Let *Y*^(*k*) ^= (*Y*^(1)^, *Y*^(2)^,..., *Y*^(*m*)^)*' *be a random m-vector and *x*^(*k*) ^= (*x*^(1)^,..., *x*^(*p*)^)*' *some explanatory variables. The conditional covariance matrix of the responses, given the explanatory variables, is represented by a factor analysis model: Σ = Ψ + BB', where Ψ is a diagonal *m × m *of uniquenesses and *B *is a *m × q *matrix of factor loadings. In the above decomposition, the diagonal elements in Ψ are referred to as the specific variances of the responses and therefore BB' appears as the shared variance in the common factor structure. This factor analysis representation of the covariance is equivalent to the following mixed effects regression modeling of the data: for *k *= 1, ..., *m*

where b_*k *_is the kth row of B, *Z *= (*Z*^(1)^, ..., *Z*^(*q*)^) are latent factors supposed to concentrate the common information in the m-responses and ϵ = (ϵ^(1)^,..., ϵ^(*m*)^)*' *is a normally distributed *m*-vector independent of *Z*, with mean 0 and variance-covariance Ψ.

An EM algorithm [11] is used to estimate Ψ, B and Z. The number of factors is chosen so that the variance of the number of false discoveries is minimized. A VARIMAX rotation is finally applied on the factors after EM estimation in order to privilege highly dispersed loadings rather than a homogeneous distribution of the loadings. Once the factor model is estimated, factor-adjusted test statistics are obtained by correction of the classical tests from the effect of the common factors. [4] show that the resulting tests statistics are asymptotically uncorrelated, which improves the overall power of the multiple testing procedure. The algorithm is implemented in the "FAMT" R package available from CRAN. For the subsequent analyses, the raw expression data set is adjusted for the estimated independent factors, which results in the so-called factor-adjusted expression data :

#### Individual and variable representation

As in PCA, the data set is transformed into a new coordinate system by an orthogonal linear transformation [12]. We can represent the individuals and variables graph through B, the matrix of factor loadings and Z, the matrix of estimated factors. Those two representations are related by a transition formula [12], which enables their simultaneous interpretation. Moreover, each factor can be related to external information which may be available in the experimental design (significance of the relationship is assessed by an analysis of variance test).

### QTL and eQTL mapping

QTLMAP software based on an interval mapping method described by Elsen *et al *[13], was used to detect QTL affecting the AF trait and the eQTL affecting the expression of DHRC7. The statistical variable for testing the presence of one QTL (or eQTL) *versus *no QTL (or no eQTL) at one location was an approximate likelihood ratio test (LRT) [14]. Significance thresholds were empirically determined for AF QTL and DHCR7 eQTL from 2000 simulations. For more details, see Le Mignon *et al *(2009) [5].

### Gene set enrichment

The enrichment of biological terms among a list of genes was assessed by the probability that an equally high or higher enrichment could be obtained by chance given the frequency of the biological terms among all the genes considered. We first implemented an R program which calculated the P value using a Fisher exact test for overrepresentation and return the enriched terms. Let  denote the subset of genes related to a given metabolism in a gene set of interest. The Fisher exact test corresponds to the hypergeometric sum as follows:  where . *B *the number of genes contained in the whole population, *m *the number of genes in the gene set of interest and *B*_0 _the number of genes related to the metabolism. The functional annotations used for this program were generated as indicated in [15] are available on the website: http://www.sigenae.org. They were obtained by a bioinformatics procedure using the Ensembl annotation source [16]. The analysis were done using the Gene ontology (GO) biological processes (BP) terms [17] and the KEGG pathways [18] with a significant threshold of 0.05.

## List of abbreviations

AF: Abdominal Fatness; eQTL: Expression Quantitative Trait Loci; GGA5: chromosome 5; FAMT: Factor Analysis for Multiple Testing; SVA: Surrogate Variable Analysis; HCA: Hierarchical Cluster Analysis; PCA: Principal Component Analysis; LRT: Likelihood Ratio Test; GO: Gene Ontology; BP : Biological Process; KEGG: Kyoto Encyclopedia of Genes and Genomes.

## Authors' contributions

GLM and SL provided the real expression data set. YB analyzed and interpreted the expression data sets supervised by SL and DC. YB carried out the QTL and eQTL mapping analyses supervised by SL. YB, SL and DC drafted the manuscript. All authors read and approved the final manuscript.

## Supplementary Material

Additional file 1 **Student test for each level of the variable "hatch"**. Student tests were performed for the levels of the variable "hatch" in order to test their effect on each factor. The crossed out column concern factors for which the global hatch effect were not significant using the Fisher test. Click here for file

Additional file 2 **Real data set: genes representation for each factor**. The genes are represented on the factor using the Z matrix of the factors found by FAMT. Click here for file
