# Supplementary figures and images for: A factor model to analyze heterogeneity in gene expression
Source: BMC Bioinformatics. 2010 Jul 2;11:368. doi: 10.1186/1471-2105-11-368 (PMC2911460; doi:10.1186/1471-2105-11-368)

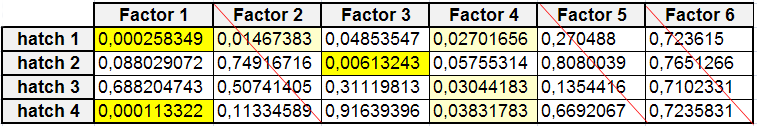

Supplement: Additional file 1 — Student test for each level of the variable "hatch". Student tests were performed for the levels of the variable "hatch" in order to test their effect on each factor. The crossed out column concern factors for which the global hatch effect were not significant using the Fisher test. [file 1471-2105-11-368-S1.PNG]

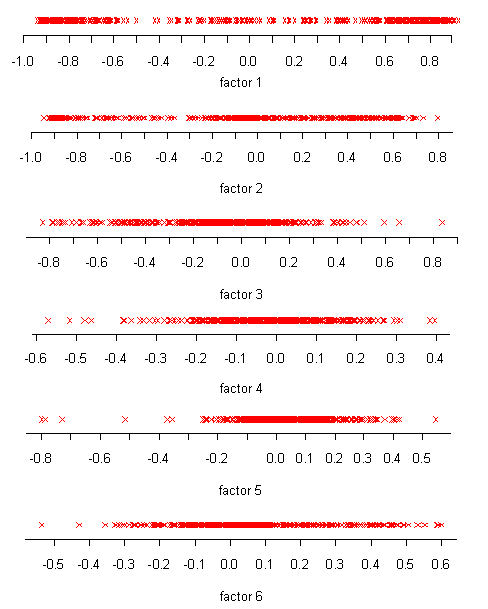

Supplement: Additional file 2 — Real data set: genes representation for each factor. The genes are represented on the factor using the Z matrix of the factors found by FAMT. [file 1471-2105-11-368-S2.PNG]
